# Characterizing the Use of Exercise Testing in Repaired Tetralogy of Fallot Patients: A Multi-Institutional Survey

**DOI:** 10.21203/rs.3.rs-3131080/v1

**Published:** 2023-07-07

**Authors:** Meghan S. Drastal, Aimee M. Layton, Michael A. Fremed

**Affiliations:** Columbia University Vagelos College of Physicians and Surgeons; Columbia University Vagelos College of Physicians and Surgeons and NewYork Presbyterian Morgan Stanley Children’s Hospital; Columbia University Vagelos College of Physicians and Surgeons and NewYork Presbyterian Morgan Stanley Children’s Hospital

**Keywords:** Cardiopulmonary Exercise Test, Pediatric, Cardiology, Tetralogy of Fallot, Aerobic Capacity, Survey

## Abstract

Long-term survival for repaired Tetralogy of Fallot (rTOF) is excellent. We achieve this by close clinical monitoring to stratify prognosis and guide clinical decision-making. Cardiopulmonary exercise stress testing (CPET) is used to help guide clinical decision making; however, there are no clear guidelines for its use in this population. We sought to describe practice variability with regards to exercise testing for rTOF patients and how exercise data is used to guide management. We distributed a survey to pediatric cardiologists via email. Analyses were performed using qualitative statistics, two-sample T-tests, and chi- squared analysis. One-hundred and three clinicians completed the survey with 83% reporting that they routinely send symptomatic rTOF patients for exercise testing and 59% for asymptomatic patients. Respondents who routinely test asymptomatic patients reported higher levels of perceived helpfulness of exercise testing (p = 0.04) and comfort with CPET interpretation (p = 0.03). Nearly all respondents (92%) reported changing management primarily based on exercise testing results, with 62% reporting “occasionally changing management” and 10% reporting “frequently changing management”. Results indicated that exercise test results influenced clinical decisions, such as the timing of interventions, need for additional imaging, or the initiation of exercise interventions. There was a statistically significant relationship between the perceived helpfulness of exercise testing and the likelihood of management changes (p < 0.01). The variability in attitudes and practices highlights the need for evidence-based guidelines addressing exercise testing in rTOF, particularly for asymptomatic patients.

## Introduction:

Congenital heart disease occurs in approximately 1% of live births worldwide [1, 2]. Right heart obstructive lesions comprise a significant portion of defects, with Tetralogy of Fallot (TOF) representing nearly 5% of all congenital heart diseases [2]. TOF patients generally undergo repair within their first year of life with the goal of balancing right ventricular outflow obstruction and pulmonary valve insufficiency.

Although long-term survival for patients with repaired tetralogy of Fallot (rTOF) is excellent, there is considerable long-term morbidity related to right ventricular volume overload from pulmonary insufficiency, including impaired exercise tolerance, arrhythmias, and heart failure [3–5]. Many rTOF patients ultimately require pulmonary valve replacement (PVR) to correct for residual pulmonary regurgitation following initial repair, though the indications for and timing of this procedure remain debated [6]. These patients are followed closely by cardiologists, who use a variety of clinical tools to guide clinical decision-making, including echocardiography, cardiac magnetic resonance imaging (MRI), and cardiopulmonary exercise testing (CPET) to assess right heart function, volume load, degree of valvar regurgitation, presence of outflow tract obstruction and/or aneurysm, and differential pulmonary blood flow [7].

Despite the ability for clinicians to use exercise testing as a tool for the non-invasive assessment of heart function under stress, there are no clear guidelines of when or how to use CPET in the clinical decision making of rTOF care. Indications for how to CPET in the broader population of patients with congenital heart diseases include: assessing physical capacity, obtaining data to support need for surgical intervention, measuring response to interventions and/or medications, assessing risk for future disease complications, and instilling confidence in a child’s ability to be physically active [8]. CPET parameters that have demonstrated a significant relationship with prognosis or indicators of morbidity and mortality within the rTOF population include decrease in peak oxygen consumption (VO_2_ peak ml/kg.min), VE/CO2 slope (ratio of minute ventilation to carbon dioxide production), and heart rate reserve [10, 11].

Despite the proposed value of exercise testing, there are currently no clear clinical guidelines for its use in the rTOF population. The lack of consensus guidelines regarding exercise testing and how to incorporate the results from this testing into clinical decision-making creates the opportunity for broad variation in clinical practice among pediatric cardiologists. To our knowledge, no previous studies have characterized current exercise testing practice patterns in the clinical setting. To better characterize this, we surveyed practicing pediatric cardiologists across several countries to determine how exercise testing is utilized in the management of rTOF patients and how they utilize data from exercise testing to guide clinical decision-making. We hypothesized that there is significant variability in provider attitudes towards exercise testing and how the data obtained impacts management. As a secondary aim, we assessed differences in practice regarding exercise testing use based on demographic information and provider attitudes. The information obtained through this survey identifies knowledge gaps in this field and emphasizes the need for evidence-based clinical guidelines for the use of exercise testing in the repaired TOF population.

## Materials and Methods:

### Survey Design and Distribution

An online survey including 29 questions was created via the electronic survey platform Qualtrics (Qualtrics International, Seattle, Washington, USA) (Supplementary Information, Online Resource 1). The purpose of the survey was to assess the utilization of exercise testing and its results in rTOF patients by pediatric cardiologists. Before distribution, the survey was reviewed and pretested by a group consisting of pediatric cardiologists and an exercise physiologist. This study was approved by the Columbia University Irving Medical Center Institutional Review Board (Protocol # AAAU2494).

The survey consisted of questions on demographic information and accessibility of exercise testing modalities. Demographics questions included information about practice location, setting, number of rTOF patients, and years in practice. Additional questions assessed utilization rates towards exercise testing in symptomatic (e.g., shortness of breath, palpitations, chest pain with exertion) and asymptomatic rTOF patients. Survey questions also addressed how results from exercise testing have changed or not changed management decisions, attitudes regarding perceived helpfulness and importance of exercise testing data, and provider comfort with CPET result interpretation. Respondents were also asked how abnormalities in metabolic parameters including maximum oxygen consumption (VO_2_ max), oxygen pulse, and VE/CO2 would impact their thinking concerning exercise, medical, and percutaneous or surgical interventions.

The survey was distributed via e-mail to the members of the American Academy of Pediatrics Section on Cardiology and Cardiac Surgery (AAP: SOCCS) and PediHeartNet, an online pediatric cardiology network. Survey responses were collected between September 2022 and November 2022. Survey responses were included in data analysis if any portion of the questionnaire beyond demographic information was completed.

### Statistical Analysis

Statistical analysis was performed using STATA version 17.0 (StataCorp, College Station, Texas, USA). Findings were primarily analyzed using standard summary statistics. Differences in responses were also compared using two-sample T-tests for continuous variables and chi-squared testing for categorical variables. Two-tailed significance was set at a p-value < 0.05.

## Results:

### Response Rates and Respondent Demographics

Surveys were opened by 118 respondents, 103 of whom completed at least some of the survey items beyond demographic information, yielding a “best case” response rate of 87% (103/118). Survey responses were collected from six different countries, with 94% (n = 97) from the United States (U.S.) and 6% (n = 6) from non-U.S. based respondents. All major geographic regions of the United States were represented. Additional demographic data are summarized in Table 1.

### Accessibility of Exercise Testing Modalities

Various exercise testing modalities were reasonably accessible to survey respondents and/or their patients. In the survey, clinicians indicated that the following tests were accessible to them: treadmill ECG/cycle ergometer with gas exchange (n = 86, 85%), treadmill ECG/cycle ergometer without gas exchange (n = 83, 82%), six-minute walk test (n = 72, 71%), and exercise stress echocardiography (n = 67, 66%). Treadmill testing (n = 73, 72%) was more frequently performed than cycle ergometry (n = 14, 14%). The distribution of testing modalities used in symptomatic patients included treadmill electrocardiogram/cycle ergometer with gas exchange (n = 68, 80%), treadmill electrocardiogram/cycle ergometer without gas exchange (n = 30, 35%), six-minute walk test (n = 8, 9%), and stress cardiac MRI (n = 7, 8%). Types of routine testing performed in asymptomatic patients was similar: treadmill electrocardiogram/cycle ergometer with gas exchange (n = 48, 79%), treadmill electrocardiogram/cycle ergometer without gas exchange (n = 19, 31%), six-minute walk test (n = 3, 5%), and stress cardiac MRI (n = 1, 2%).

### Frequency of Routine Testing

With regards to routine exercise testing practices, 83% of respondents reported routinely sending symptomatic patients for diagnostic exercise testing while only 59% of respondents reported routinely sending asymptomatic rTOF patients for medical surveillance (n = 61). Those who routinely send asymptomatic patients predominantly send patients who underwent trans-annular patch (n = 58, 95%) but less frequently send those who underwent valve-sparing repair (n = 36, 59%), right ventricle to pulmonary artery (RV-PA) conduit (n = 49, 80%), and pulmonary valve replacement (n = 49, 80). The age at which these pediatric cardiologists typically start testing asymptomatic patients varied, with 10% referring at younger than 10 years (n = 6), 36% between 10 to 12 years (n = 22), 36% between 13 to 15 years (n = 22), 10% between 16 to 17 years (n = 6), and 8% at 18 years or older (n = 5). Most providers reported performing exercise testing every 2 to 3 years or every 4 to 5 years with a small portion sending patients annually (Table 2).

Compared to testing in asymptomatic patients, there was less variability in practice regarding routine exercise testing in symptomatic rTOF patients (n = 86, 83%). Of the respondents that routinely send symptomatic patients for exercise testing, the majority send patients annually or every 2 to 3 years, with a small portion sending patients every 6 months or every 4 to 5 years (Table 2). There was a statistically significant difference in frequency of routine testing in asymptomatic and symptomatic with more frequent testing in symptomatic patients.

### Provider Attitudes on Exercise Testing

**Figure 1:**
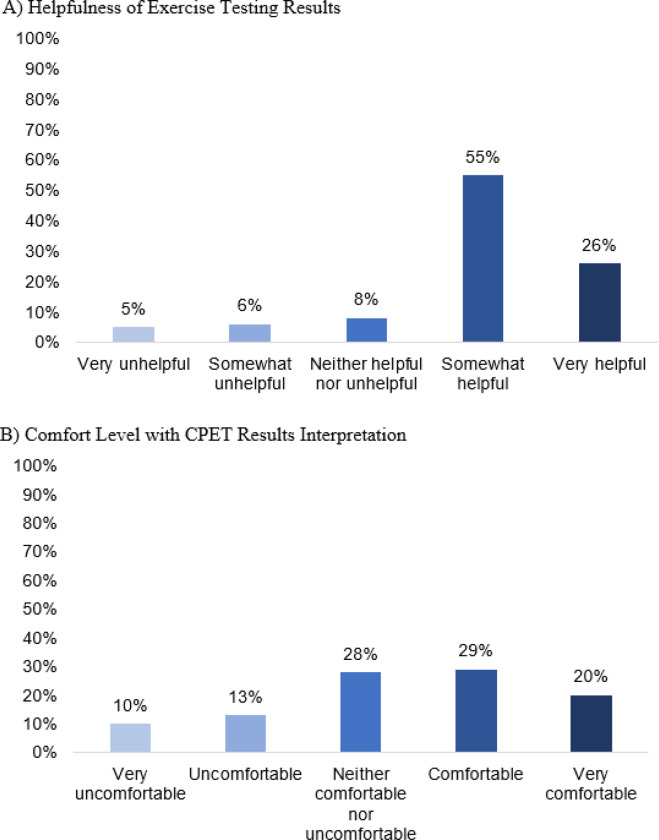
Provider Attitudes Regarding Perceived Helpfulness of Exercise Testing (A) and Comfort with CPET Data Interpretation (B)

**Figure 2:**
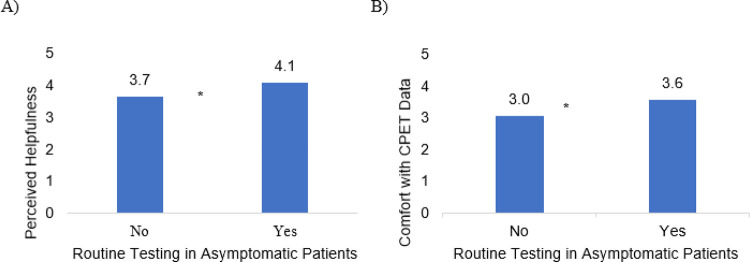
Effect of Attitudes Regarding Perceived Helpfulness (A) and Comfort with CPET Data Interpretation (B) on Routine Testing Practices in Asymptomatic Patients (*= p<0.05)

Most respondents viewed exercise testing as somewhat helpful in guiding their clinical decision making (n = 55, 55%). Twenty-six percent of practitioners stated they found it very helpful (n = 26, 26%), while 11% (n = 11) stated it was not unhelpful in guiding their clinical decision making (Fig. 1). While exercise testing was largely considered to be helpful, there was more heterogeneity with regards to comfort with CPET interpretation with 20% reporting “very comfortable” (n = 20), 29% “comfortable” (n = 29), 28% as “neither comfortable nor uncomfortable” (n = 28), 23% “uncomfortable” or “very uncomfortable” (n = 23) (Fig. 1). There was a statistically significant relationship between comfort with CPET test interpretation and perceived helpfulness of exercise testing (p < 0.01). Respondents who routinely test asymptomatic patients reported higher levels of perceived helpfulness of exercise testing (p = 0.04) and comfort with CPET interpretation (p = 0.03) when compared to those who do not routinely test asymptomatic patients (Fig. 2). There were no statistically significant differences in comfort with interpretation of CPET results or perceived helpfulness with regard to practice setting or years of experience.

### Impact of Exercise Testing Results on Management Changes

**Figure 3:**
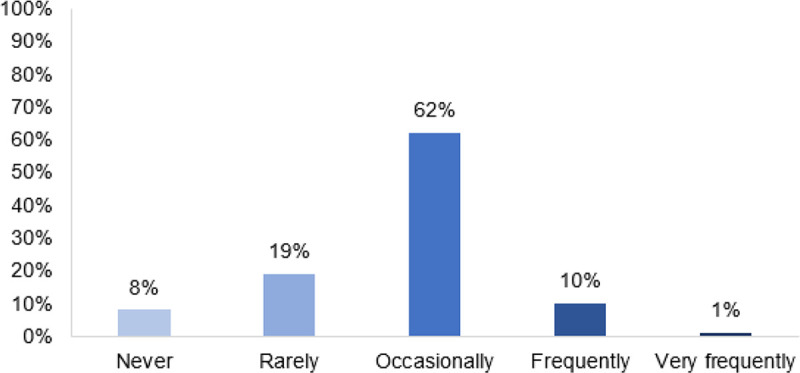
Frequency of Reported Management Changes Based on Exercise Testing Results

Nearly all respondents (n = 92, 92%) reported changing management primarily based on exercise testing, with 62% (n = 62) reporting “occasional” and 10% (n = 10) reporting “frequent” management changes (Fig. 3). Of the respondents that reported changing management primarily in response to exercise testing, types of management changes involved expediting (n = 67, 67%) or delaying (n = 29, 29%) referral for surgical or percutaneous intervention, obtaining additional imaging (n = 43, 43%), or prescribing exercise intervention (n = 35, 35%) (Table 3).

The majority of survey respondents report providing routine exercise counseling for their rTOF patients (n = 85, 85%), with 49% reporting “occasional” (n = 42) and 14% reporting “frequent” (n = 12) changes to counseling based on exercise testing results. Over 80% of survey respondents (n = 68) believe that exercise testing should be standard of care for patients with rTOF. There was a statistically significant relationship between the perceived helpfulness of exercise testing and the likelihood of management changes (p < 0.01). There was no statistically significant relationship between comfort of ability to interpret test results with CPET interpretation and the likelihood of management changes.

**Figure 4:**
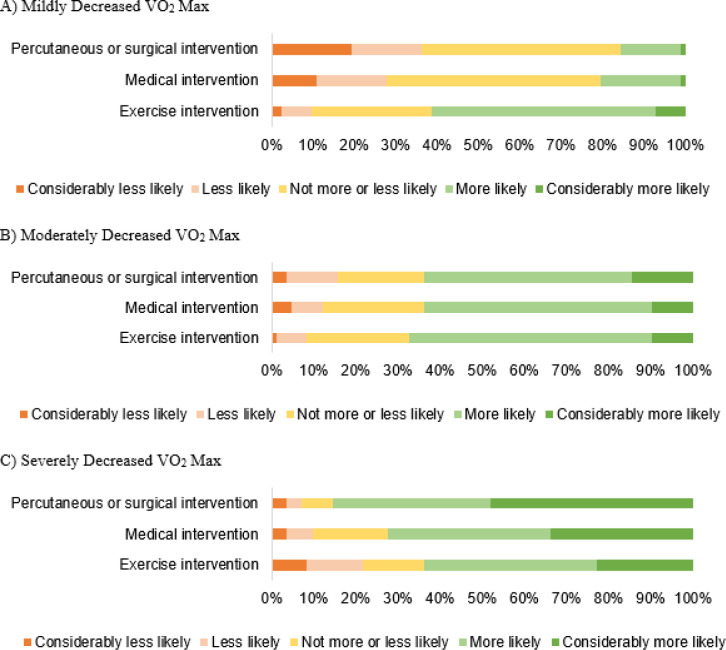
Scenarios Assessing Likelihood of Management Decisions Based on Mildly Decreased VO_2_ Max (A), Moderately Decreased VO_2_ Max (B), and Severely Decreased VO_2_ Max (C)

Respondents were increasingly more likely to consider percutaneous/surgical or medical interventions in response to mild, moderate, and severe decreases in VO_2_ max (Fig. 4).

## Discussion:

The results of our study demonstrate that clinicians working with the rTOF patient population frequently rely on CPET results to guide clinical decision-making. However, there was considerable variability in the frequency of testing. There was also variability in how the clinicians used the test results to inform what clinical intervention to use. The majority of clinicians use the test results to determine timing for percutaneous or surgical intervention, despite no known guidelines on thresholds or specific parameters to follow to guide such decisions. These results highlight the need for clinical guidelines or possibly more research to determine how exercise test results should be used in the clinical care of the rTOF population.

While recent recommendations support the use of exercise testing in pediatric congenital heart disease patients, guidance is generally non-specific without substantial details on recommended practices, such as patient selection or frequency [12]. In one recent study, Goldmuntz et al. demonstrated variation in practice patterns concerning the general management of Tetralogy of Fallot patients in the ambulatory setting [13]. To our knowledge, this is the first multi-institutional study characterizing the use of exercise testing in rTOF patients. These data are important in justifying the need for expanded clinical guidelines, or potentially further research, in how exercise testing relates to clinical outcomes in the rTOF patient population.

Our study demonstrates that nearly all surveyed clinicians routinely perform exercise testing in symptomatic rTOF patients. There is considerably more variation in the use and frequency of exercise testing of surveillance of disease progression of asymptomatic patients. Despite the evidence-based utility of exercise testing in the objective assessment of exercise capacity in asymptomatic patients [14], only 59% of clinicians regularly exercise test their asymptomatic patients. Existing guidelines generally do not specify recommended practices based on symptom burden, yet our survey identified this as a key point of divergence among pediatric cardiologists.

There are several possible explanations for the observed variation in clinical practice, including personal, patient, institutional, and/or financial reasons. Interestingly, pediatric cardiologists that reported higher levels of comfort with CPET interpretation were significantly more likely to routinely test asymptomatic patients. This suggests that training and post-fellowship practice can influence attitudes and use of exercise data. Further, the degree of perceived helpfulness of exercise data was related to provider comfort with interpretation of exercise data, again supporting that training plays a major role in how the test results are used. Survey respondents with a higher level of comfort with CPET interpretation were significantly more likely to find exercise testing helpful. While these results may not be surprising, they do highlight the need for increased education on exercise testing modalities, including CPET, during cardiology training. While we hypothesized that there would be differences based on experience level, we did not find any statistically significant differences in routine testing practices, perceived helpfulness, or CPET comfort level based on number of years in practice. Until recently, exercise testing has made up a relatively small component of pediatric cardiology fellowship curricula despite the growing recognition of the importance of the field. In 2022 the Pediatric Cardiology Exercise Medicine Curriculum Committee (PCEMCC) was created to set forth exercise physiology training recommendations [15]. This is the first significant attempt to boost training in exercise testing, which may account for the absence of observable differences based on experience level in our survey. Nevertheless, the renewed focus on exercise testing in pediatric cardiology fellowship should lead to increased comfort among providers with interpretation of exercise data and can help guide standardization of exercise testing practices in the future.

Another key finding of this survey was that almost all survey respondents indicated that they have altered management based on exercise testing results. Although management decisions must consider the complete clinical picture including history, physical exam, and additional diagnostic workup, exercise testing appears to play an important role in clinical decision-making. In particular, these results may be helpful when deciding on the timing of interventions, the need for additional imaging, or the initiation of exercise interventions. The most frequent type of management change cited among pediatric cardiologists was referral for surgical or percutaneous interventions. Following repair, Tetralogy of Fallot patients generally have an incompetent pulmonary valve with varying degrees of residual pulmonary stenosis with resultant hemodynamic consequences [16]. These residual lesions often require surgical or percutaneous intervention; however, the ideal timing for reintervention remains challenging [6]. While there is recognized prognostic value of CPET, there remains significant uncertainty surrounding the use of CPET to determine the timing of PVR [7, 17]. As demonstrated by these survey results, exercise testing may serve as a valuable adjunct to current risk stratification strategies when assessing the need for surgical or percutaneous interventions. However, further research is needed to further define the optimal role of exercise testing in management decisions, particularly those involving surgical/percutaneous interventions. Additionally, in future survey studies, it would be helpful to delineate the specific types of surgical/percutaneous interventions that providers are referring patients to in response to exercise testing findings. Interestingly, survey respondents also indicated that exercise testing plays a role in the decision to delay referrals for surgery/percutaneous interventions. An encouraging exercise test in the setting of an otherwise unremarkable workup may provide additional reassurance to providers and potentially reduce unnecessary testing and/or interventions.

The survey also sought to determine how specific CPET abnormalities might impact the likelihood of the provider making certain management decisions. There was considerable variation in the likelihood of management decisions based on the specified parameters: mildly decreased VO_2_ max, moderately decreased VO_2_ max, severely decreased VO_2_ max, decreased minute ventilation/carbon dioxide production ratio (VE/VCO2) slope, and early plateau or drop in oxygen pulse. Surveyed pediatric cardiologists appeared to be most likely to intervene in the case of a severely decreased VO_2_ max in contrast to the other parameters tested. A possible explanation for these findings is that VO_2_ max is more well-established than other CPET parameters, and providers may not be as familiar or comfortable with other parameters and would therefore not make management decisions based on them.

There are several important limitations to the current study. As with any survey-based study, results only reflect the opinions and practices of those who return responses, creating potential for selection bias. It is possible that respondents who do not routinely use exercise testing chose not to participate in the study, given that it was the topic of interest for the survey; however, there was still a considerable percentage of respondents who indicated they did not routinely perform exercise testing. Recall bias is also inherent to any survey-based research though only a small percentage of the survey items required recall. Finally, though we collected data about access to testing modalities, we did not critically assess the various barriers to testing that may be present. We also did not specifically ask about makeup of lab personnel; however, the presence of an exercise physiologist likely impacts the utility of CPET. Future studies that further characterize barriers to testing access, such as structural, financial, and patient-specific factors, would be beneficial.

In conclusion, this study provides an overview of the current landscape of exercise testing in rTOF patients and highlights the current variations in practice patterns among pediatric cardiologists, particularly in asymptomatic patients. Exercise testing plays an important role in managing rTOF patients; however, there remains considerable uncertainty surrounding optimal testing practices. These findings emphasize the need for evidence-based, consensus guidelines detailing the use of exercise testing in the management of rTOF patients and support the current efforts of fellowship program directors to increase exposure to exercise testing during training.

## Figures and Tables

**Table 1:** Respondent Demographics

Demographic Information	n	%
*Region*		
New England	6	6%
Mid-Atlantic	21	22%
Southeast	20	21%
Midwest	33	34%
Southwest	6	6%
West	11	11%
*Practice Settings*		
Academic Medical Centers	77	75%
Community Hospitals	6	6%
Private Practices	7	7%
Combination of Settings	12	12%
Other	1	1%'
*Years of Experience*		
Still in Fellowship	4	4%
< 5 years	19	18%
5–10 years	20	19%
11–15 years	17	17%
16–20 years	6	6%
>20 years	37	36%

**Table 2:** Frequency of Routine Exercise Testing in Symptomatic vs. Asymptomatic Patients

Frequency of Routine Testing	Asymptomatic Patients % (n)	Symptomatic Patients % (n)	p value
Every 6 months	0	4 (3)	
Annually	3 (2)	55 (47)	0.580
Every 2 to 3 years	72 (44)	38 (32)	0.010
Every 4 to 5 years	25 (15)	4 (3)	0.092

**Table 3:** Types of Management Changes Made Primarily Based on Exercise Testing Results

Type of Management Change	n	%
Refer to surgery or percutaneous intervention	67	67%
Refer for cardiac catheterization	51	51%
Obtain additional imaging	43	43%
Repeat exercise testing sooner than originally planned	38	38%
Prescribe exercise or rehabilitation intervention	35	35%
Delay referral for surgery or percutaneous intervention	29	29%
Delay office visit beyond originally scheduled date	9	9%
Other:	5	5%
